# A System Dynamics Approach to Model Photosynthesis at Leaf Level Under Fluctuating Light

**DOI:** 10.3389/fpls.2021.787877

**Published:** 2022-01-28

**Authors:** Nicole Salvatori, Fabrizio Carteni, Francesco Giannino, Giorgio Alberti, Stefano Mazzoleni, Alessandro Peressotti

**Affiliations:** ^1^DI4A, Department of Agri-Food, Environmental and Animal Sciences, University of Udine, Udine, Italy; ^2^Department of Life Sciences, University of Trieste, Trieste, Italy; ^3^Department of Agricultural Sciences, University of Naples Federico II, Portici, Italy; ^4^Faculty of Science and Technology, Free University of Bolzano, Bolzano, Italy; ^5^Task Force on Microbiome Studies, University of Naples Federico II, Naples, Italy

**Keywords:** modeling, photosynthesis, Chl-deficient, soybean, system dynamics

## Abstract

Photosynthesis has been mainly studied under steady-state conditions even though this assumption results inadequate for assessing the biochemical responses to rapid variations occurring in natural environments. The combination of mathematical models with available data may enhance the understanding of the dynamic responses of plants to fluctuating environments and can be used to make predictions on how photosynthesis would respond to non-steady-state conditions. In this study, we present a leaf level System Dynamics photosynthesis model based and validated on an experiment performed on two soybean varieties, namely, the wild type Eiko and the chlorophyll-deficient mutant MinnGold, grown in constant and fluctuating light conditions. This mutant is known to have similar steady-state photosynthesis compared to the green wild type, but it is found to have less biomass at harvest. It has been hypothesized that this might be due to an unoptimized response to non-steady-state conditions; therefore, this mutant seems appropriate to investigate dynamic photosynthesis. The model explained well the photosynthetic responses of these two varieties to fluctuating and constant light conditions and allowed to make relevant conclusions on the different dynamic responses of the two varieties. Deviations between data and model simulations are mostly evident in the non-photochemical quenching (NPQ) dynamics due to the oversimplified combination of PsbS- and zeaxanthin-dependent kinetics, failing in finely capturing the NPQ responses at different timescales. Nevertheless, due to its simplicity, the model can provide the basis of an upscaled dynamic model at a plant level.

## Introduction

The continuous rise in population requires an increase in agricultural production of at least 60% (Alexandratos and Bruinsma, 2012). By being the source of food and responsible for the survival of the majority of life on Earth (Stirbet et al., 2020), photosynthesis has recently become a target to improve global food production, since the increase in genetic yield potential seems to be hindered (Foyer et al., 2017; Taylor and Long, 2017). Photosynthesis has been intensively studied in laboratories, but, due to its complex nature, it still provides some challenges (Flexas et al., 2012). Mathematical models can provide a different tool to better understand the dynamics of this process and can be used to make predictions on how photosynthesis would respond to limiting situations (Stirbet et al., 2020).

Several modeling efforts have been done in order to describe photosynthesis as a whole. The models can be differentiated by considering the processes at steady-state (Farquhar et al., 1980; Buckley et al., 2003; Ye et al., 2013) or non-steady-state (Kirschbaum et al., 1997; Morales et al., 2018; Bellasio, 2019; Nedbal and Lazàr, 2021); for their spatial scale, i.e., leaf scale (Farquhar et al., 1980; Zhu et al., 2013; Vialet-Chabrand et al., 2017a) or canopy scale (Song and Zhu, 2012); and for the different modeling approaches, i.e., empirical models (Vialet-Chabrand et al., 2017a), system biology models (Pettersson and Ryde-Pettersson, 1988; Zhu et al., 2013; Kannan et al., 2019), and process-based models (Kirschbaum et al., 1997; Morales et al., 2018; Bellasio, 2019).

The processes of photosynthesis have been initially tackled simulating steady-state conditions (Farquhar et al., 1980; Von Caemmerer, 2013). These models are fundamental in understanding the physiological characteristics and answering very specific questions, but usually overestimate total photosynthesis in fluctuating environmental conditions (Timm et al., 2004). In fact, external conditions are rarely stable in natural environments, so plants need to continuously adjust to optimize the carbon uptake in these dynamic conditions (Kaiser et al., 2018). Different adjustments can be operated by plants depending on the time scale considered (Kono and Terashima, 2014): in the fast temporal scale, plants respond by regulating the mechanisms involved in photochemical (Kono and Terashima, 2014; Kaiser et al., 2018) and non-photochemical processes (Acebron et al., 2021), activating the Calvin Cycle enzymes (Porcar-Castell et al., 2006) and moving their chloroplasts within the leaves (Kaiser et al., 2015); slower adjustments can then be due to the regulation of the stomata (Buckley, 2017; Vialet-Chabrand et al., 2017b; Matthews et al., 2018), the movements of the leaves within the canopy (as photonastic movements or due to the wind), and the adaptative adjustments in nitrogen and chlorophyll content (Posada et al., 2009; Zhang et al., 2016).

Among the main variable conditions, the most relevant is light. Light intensity is continuously changing due to the movements of the clouds and to the wind moving the leaves (Pearcy, 1990; Retkute et al., 2015). Plants need to adapt to these changes in light conditions, and some species may be more efficient than others in doing it (Kromdijk et al., 2016; Urban et al., 2017; Matsubara, 2018). One rising question is if a reduction in chlorophyll content might be detrimental or beneficial when dealing with fluctuating light conditions. At a canopy level, the role of chlorophyll content has been investigated (Ort et al., 2015; Slattery et al., 2016; Gu et al., 2017; Walker et al., 2018), and it has been proposed that a reduced chlorophyll content would entail a better distribution of the light in the lower layers of the canopy, therefore increasing the overall photosynthesis. Nevertheless, few studies have analyzed the effect of chlorophyll reduction in fluctuating environments (Ferroni et al., 2020).

In this study, we focused on the effect of fluctuating light on two soybean varieties, namely, the green wild type soybean (Eiko) and a chlorophyll-deficient mutant (MinnGold), which has been first described by Campbell et al. (2015). It has been shown that MinnGold has comparable light curves and A/Ci curves (steady-state measurements) at leaf level compared with Eiko, but lower biomass was found at harvest (Sakowska et al., 2018). It was hypothesized that a slower adjustment to fluctuating light might cause a lower carbon accumulation at a canopy level and that steady-state measurements at leaf level would not be able to capture this difference (Genesio et al., 2020).

Therefore, in this study, we reported the investigation of the role of the chlorophyll content in adjusting to light fluctuations, combing experimental observations with a modeling framework. To begin with, we implemented a model at leaf level to be a basis in understanding the response of these two varieties to highly fluctuating light environments. We decided to use a process-based approach, based on the principles of System Dynamics, according to which a complex system can be represented by flows, compartments (stocks), and feedback loops (Forrester, 1971).

## Materials and Methods

Two soybean varieties have been used in this study with different chlorophyll contents, namely, Eiko, the green cultivar used as the wild type and MinnGold, the chlorophyll-deficient mutant (Campbell et al., 2015; Sakowska et al., 2018). The plants were sown in 3 L pots and grown inside a controlled growth chamber system (Salvatori et al., 2021) for 5 weeks with either non-fluctuating light or fluctuating light conditions. The light was turned on from 5:00 to 19:00 h, and the intensity was set to simulate the daily profile of the sun, reaching a maximum of 650 μmol m^–2^ s^–1^ for the non-fluctuating light protocol or fluctuating every minute (1 min high-light and 1 min low-light) with an amplitude of ± 20% around the non-fluctuating light intensity value (Supplementary Figure 1). By doing this, all plants received the same amount of light throughout the day. In each chamber, we placed a ceptometer at the level of the pots to continuously measure the transmitted light (tPPFD); we also measured the albedo every 2–3 days. Therefore, by knowing the incident light (PPFD), we estimated the absorbed light (aPPFD) by the canopy as aPPFD = PPFD − rPPFD − tPPFD, where rPPFD = PPFD ⋅ albedo. Therefore, the absorption coefficients (α = aPPFD/PPFD) were estimated as 0.78 and 0.54 for Eiko and MinnGold, respectively.

Next, three plants from each variety and each light protocol were randomly chosen as replicates, from which we selected a young and fully expanded leaf to perform fluorescence analysis combined with gas exchange using the LI-6800 (Licor Biosciences, Nebraska, United States) equipped with infrared gas analyzers (IRGA) coupled with pulse-amplitude modulation (PAM) fluorometer. In particular, we were interested in recording the carbon assimilation (*A*), the electron transport rate (*ETR*), and the non-photochemical quenching (*NPQ*).

We used the following protocol: all plants were dark-adapted overnight, then the light was turned on following either a constant light protocol for 60 min at 650 μmol m^–2^ s^–1^ or a fluctuating light protocol with light intensity changing from 780 to 520 μmol m^–2^ s^–1^ every minute by simulating the growth conditions. These levels of light intensity were chosen to avoid saturating photosynthetic photon flux (Sakowska et al., 2018) in order to prevent photoinhibition. The CO_2_ levels were maintained at 400 ppm, vapor pressure deficit (VPD) was kept at 1.8 kPa, and leaf temperature was kept at 25°C.

The carbon assimilation rate (A in μmol CO_2_ m^–2^ s^–1^) was calculated as follows:


$$A=\frac{\mu_{0}\left[c_{0}-c_{a}\left(\frac{1-w_{0}}{1-w_{a}}\right)\right]}{s}$$

where μ_0_ is the flow rate (μmol air s^–1^) entering the leaf chamber, *s* is the leaf area (m^2^), *c_0_* and *w_0_* are the CO_2_ and H_2_O concentrations (in μmol CO_2_ and mmol H_2_O, respectively) entering the leaf chamber, and *c_a_* and *w_a_* are the CO_2_ and H_2_O concentrations exiting the chamber.

Throughout the protocol, a saturating light pulse of > 5,000 μmol m^–2^ s^–1^ was given to the leaf sample for 800 ms every 30 s, to quantify maximal fluorescence in the light ($F_{m}^{\prime }$) and dark (*F_m_*). Each of these flashes lasts 0.8 s and added 20% to the total flux seen by the plants, arriving at an actual average illumination of approximately 800 μmol m^–2^ s^–1^. The operating efficiency of the PSII (Φ_*PSII*_) was calculated as follows (Genty et al., 1989):


$$\Phi_{PSII}=\frac{\left(F_{m}^{\prime }-F_{s}\right)}{F_{m}^{\prime }}$$

where *F_s_* is the steady-state fluorescence.

*NPQ* was calculated using the equation from Bilger and Björkman (1990) based on the Stern-Volmer method, as follows:


$$NPQ=\frac{\left(F_{m}-F_{m}^{\prime }\right)}{F_{m}^{\prime }}$$

Finally, the ETR was calculated based on Krall and Edwards (1992), as follows:


$$ETR=I\cdot \alpha \cdot fraction_{PSII}\cdot \Phi_{PSII}$$

where *I* is the incident light, *fraction*_*PSII*_ is the fraction of absorbed light that is received by the PSII and is normally set to 0.5 (Baker, 2008), α is the absorbance coefficient which was set to 0.55 for MinnGold and 0.78 for Eiko as previously shown.

### Model Description

**FIGURE 1:**
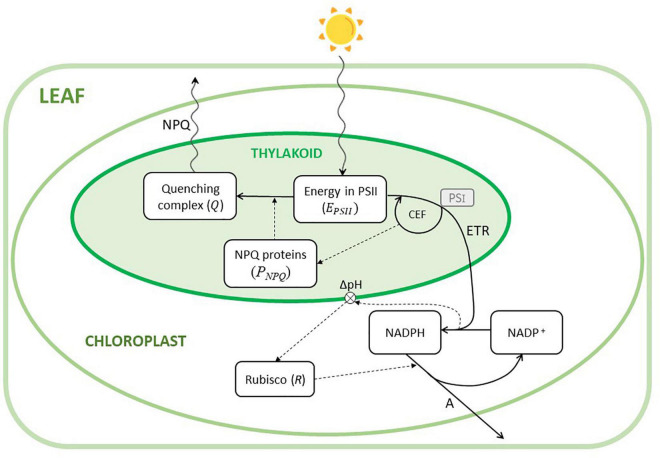
Conceptual diagram of the model. The model describes the phenomena occurring in a chloroplast. The six state variables (i.e., E_PSII_,Q,*P*_*NPQ*_,*NADPH*,*NADP*,R) are depicted by the white boxes. Continuous lines are fluxes, dashed lines describe influences, and wavy lines indicate either incident light or heat fluxes (NPQ). CEF is the cyclic electron flow transporting back electrons from photosystem I (PSI, not explicitly included in the model, represented in the small gray box) into the linear electron transport (ETR), whose energy is exploited to generate ATP (not represented) and influencing thylakoid pH and, therefore, the activation of the NPQ-related proteins (*P*_*NPQ*_). The transmembrane ΔpH is influenced by the ratio NADPH/NADP^+^, which activates Rubisco. Rubisco is then responsible for the assimilation of CO_2_ (A).

In this study, we presented a model of the main processes involved in the regulation of the photosynthesis of leaves of C3 plants exposed to fast changes of light intensity. Figure 1 shows a schematic diagram of the model structure with a simplified representation of the implemented processes, within the complex phenomena occurring in photosynthesis.

For the sake of simplicity, the model essentially considers the main dynamics of a single chloroplast as representative of a whole leaf, in a sort of “big chloroplast” approach. We did not explicitly model the limitations due to stomatal conductance. This assumption is reasonable since the modeled leaf is exposed to optimal conditions of CO_2_ and average light intensity. Even if this dynamic becomes relevant during the induction phase (dark-light transition), it is known that, in soybean, this limitation can be mainly attributed to Rubisco activation (Soleh et al., 2016, 2017; Taylor and Long, 2017). Furthermore, the two varieties did not show any statistical differences in the dynamic of the stomatal conductance (Supplementary Figure 2).

Here, we focused on the limitations imposed by the light reactions. In fact, rapid light fluctuations would not have a direct drastic effect on the Calvin-Benson cycle, because the activation/deactivation of the enzymes under fluctuations of light takes several minutes (Yamori, 2016) and, therefore, the activation level would depend on the average intensity, independently from fluctuations shorter than a few minutes. Furthermore, in low light, photosynthesis is restricted by the light absorption of the light harvesting systems (Long and Bernacchi, 2003; Li et al., 2021). When light excites the photosystem 2 (PSII), many pigments (chlorophyll *a* and *b* antenna proteins) collect this energy and transfer it to the reaction center. This number of pigments can be variable from plant to plant and determine the ability of the photosystem to transfer this energy. PSII oxidizes water to O_2_ releasing protons into the lumen. The electrons are then passed on to the Cytochrome b6f (Cyt b6f) which delivers them to photosystem 1 (PSI) transporting additional protons into the lumen. This proton pumping from the stroma into the lumen creates a pH gradient (ΔpH) (Flexas et al., 2012). For simplicity, these last processes involving Cyt b6f and PSI are not included in the model (even if their role at steady-state has been established; Johnson and Berry, 2021) and, therefore, not represented in Figure 1.

The energy transported is used to reduce the final acceptor NADP^+^ to NADPH. The ΔpH generated is then used by the ATP synthase to produce ATP as protons diffuse back from the lumen to the stroma. This process is generally called linear electron flow (LEF; in Figure 1 defined as ETR), and the chemical energy produced (ATP and NADPH) is used in the Calvin Cycle to fix CO_2_. The Calvin Cycle is regulated by the enzyme Rubisco, which is itself activated by the changing in the ΔpH generated by the electron transport. Generally, the rate of CO_2_ fixation at a steady-state has been described by the model of Farquhar et al. (1980) and Farquhar and Von Caemmerer (1982) as the minimum of the rate of carboxylation under the limitation of Rubisco activity and of RuBP regeneration. In this work, the limitation by Rubisco is explicitly included, whereas the limitation of RuBP regeneration is assumed as proportional to the rate of oxidation of NADPH to NADP^+^.

When there is an excess of energy, this can be dissipated through several processes, which are called NPQ. These mechanisms involve several processes that are differentiated on the time scale of their relaxation kinetics (Müller et al., 2001): a fast phase is assigned to qE (i.e., energy-dependent quenching), which relaxes within seconds to minutes (Krause et al., 1982); two middle phases qZ (i.e., zeaxanthin-dependent component) detectable within 10–15 min (Nilkens et al., 2010) and qT (i.e., state transition quenching) in 30 min and a slow phase qI in the time scale of an hour (Kohzuma and Hikosaka, 2018).

We only modelled the energy-dependent quenching (qE) since it is the most important component of NPQ when regarding fluctuating irradiance; in fact, by operating in the scale of minutes (Ebenhöh et al., 2014), it responds most quickly to changes in light intensities (Kaiser et al., 2015). qE is regulated by luminal pH and the xanthophyll cycle pigments. The saturation of the dark reactions causes a decrease in the luminal pH, causing the protonation of some PSII proteins (PsbS proteins) (Matuszyńska et al., 2016), the release of violaxanthin molecules and their de-epoxidation to antheraxanthin and zeaxanthin. Zeaxanthin then binds to PSII proteins (in which PsbS proteins have been protonated), forming a quenching complex favoring the dissipation of the excitation energy as heat (Porcar-Castell et al., 2006). PsbS dynamics are more relevant in the fast fluctuations of light, whereas zeaxanthin activation is related to the induction phase of photosynthesis. For the sake of simplicity, we did not distinguish between the two former processes, which have been combined. Furthermore, the generation of a ΔpH is necessary under environmental stressful conditions, when the dark reactions are saturated, allowing the production of ATP without the reduction of NADP^+^ (Roach and Krieger-Liszkay, 2014). In such cases, the cyclic electron flow (CEF) around the PSI is activated, increasing electron transfer from PSI to the plastoquinone pool, and again to PSI *via* the Cyt b6/f complex (Yamori, 2016). In C3 plants, CEF is considered negligible at steady-state conditions, thus becoming relevant under specific stressful conditions such as low CO_2_, high light, drought, or during the dark-to-light transitions (Rochaix, 2011). CEF then becomes a regulator of NPQ and ETR at non-steady-state conditions (Roach and Krieger-Liszkay, 2014; Yamori, 2016).

### Mathematical Formulation of the Model

In the model, the described processes are represented as a set of differential equations representing the dynamics of 6 state variables: the energy in PSII (*E*_*PSII*_), the activation level of the NPQ-related proteins (*P*_*NPQ*_), the quenching complex of *P*_*NPQ*_ with the PSII (*Q*), the dynamics of NADPH and NADP^+^, and the activation level of Rubisco (*R*) (Figure 1).

The first variable represents the excitation energy of PSII. When the photosystem receives a light input, the excitation energy is transferred either as linear electron transport (ETR), regulated by the amount of the final acceptor NADP^+^ or as dissipation of energy, regulated by *P*_*NPQ*_. *P*_*NPQ*_ combines the dynamics of the zeaxanthin activation and the PsbS protonation. The following equation describes these processes:


(1) $$\frac{dE_{PSII}}{dt}=\overset{Energyinput}{\overbrace{\alpha \cdot c_{in}\cdot PAR\cdot \left(1-\frac{E_{PSII}}{E_{PSII}^{*}}\right)}}-\overset{ETR}{\overbrace{v_{ETR}\cdot E_{PSII}\cdot NADP^{+}}}-\overset{Energydissipation}{\overbrace{v_{d}\cdot E_{PSII}\cdot P_{NPQ}\cdot \left(1-\frac{Q}{Q^{*}}\right)}}$$

The energy input is formulated as a logistic equation—since the photosystem can hold a maximum amount of energy ($E_{PSII}^{*}$) that depends on the amount of chlorophylls present—with a velocity of induction *c*_*in*_ which is linearly related to the absorption coefficient (α) and the photosynthetically active radiation (*PAR*) (Table 1). The linear electron transport is linearly related to the energy in PSII (*E*_*PSII*_) and to the amount of NADP^+^ in the chloroplast stroma, with a velocity of induction *v*_*ETR*_.

**TABLE 1:** State variables, fixed parameters, and calibrated parameters of the model.

	Symbol	Description	Units	Value		
				Eiko		MinnGold
State variables	*E*_*PSII*_	Energy in photosystem II (*t* = 0)	μmol m^–2^	0		
	*Q*	Quenching complex (*t* = 0)	μmol m^–2^	0		
	*P*_*NPQ*_	Activation level of the NPQ-related proteins (*t* = 0)	–	0		
	*NADP* ^+^	NADP^+^ in chloroplast stroma (*t* = 0)	–	5		
	*NADPH*	NADPH in chloroplast stroma (*t* = 0)	–	5		
	*R*	Rubisco activation level (*t* = 0)	–	0.001		
Fixed parameters	*PAR*	Photosynthetically active radiation	μmol m^–2^s^–1^	520 or 780		
	α	Absorption coefficient	–	0.78		0.54
Calibrated parameters	*c*_*in*_	Energy input coefficient	–	0.23		0.25
	$E_{PSII}^{*}$	PSII energy carrying capacity	μmol m^–2^	157.56		9.98
	*v*_*ETR*_	Velocity of ETR	s^–1^	0.78		11.56
	*v_d_*	Velocity of energy dissipation	s^–1^	0.08		7.00
	Q*	PSII-zeax complex energy carrying capacity	μmol m^–2^	0.07		0.03
	*v*_*NPQ*_	Velocity of NPQ	s^–1^	70.58		53.87
	*v_p_*	Maximum velocity of NPQ-related proteins activation	s^–1^	0.07		0.01
	*v_C_*	Maximum velocity of Calvin Cycle reactions	s^–1^	11.75		13.04
	η_*NADPH*_	Efficiency of NADPH	–	5.07		4.10
	η_*NADP*^+^_	Efficiency of NADP^+^	–	0.89		0.75
	*v_R_*	Maximum velocity of Rubisco activation	s^–1^	8.9⋅10^–4^		14⋅10^–4^
	*d*	Maximum ΔpH balance value	–	8.40		3.69
	*c_y_*	Minimum necessary cyclic electron flow	–	–4		0

Then the excess energy in PSII can be dissipated only if zeaxanthin has formed the quenching complex with the PSII protonated (*Q*). This complex is then able to release energy as heat (*NPQ*). The dynamics of the quenching complex of *P*_*NPQ*_ with PSII are described as follows:


(2) $$\frac{dQ}{dt}=\overset{Energydissipation}{\overbrace{v_{d}\cdot E_{PSII}\cdot P_{NPQ}\cdot \left(1-\frac{E_{z}}{E_{Z}^{*}}\right)}}-\overset{NPQ}{\overbrace{v_{NPQ}\cdot Q}}$$

The energy dissipation flux has an upper limit described by the saturation term of the quenching complex (1−Q/Q*) and a velocity of induction *v_d_*. The energy released as heat (*NPQ*) is then linearly related to *Q* with a velocity of induction *v*_*NPQ*_.

The activation level of the *P*_*NPQ*_ is then related to the cyclic electron transport (*CEF*) as follows:


(3) $$\frac{dP_{NPQ}}{dt}=\left\{ \begin{array}{cc} \overset{Proteinactivation}{\overbrace{v_{p}\cdot \left(1-P_{NPQ}\right)}} & ifCEF>c_{y}\\ 0 & ifCEF\leq c_{y} \end{array}\right.$$

with


$$CEF=\overset{Energyinput}{\overbrace{\alpha \cdot c_{in}\cdot PAR\cdot \left(1-\frac{E_{PSII}}{E_{PSII}^{*}}\right)}}-\overset{ETR}{\overbrace{v_{ETR}\cdot E_{PSII}\cdot NADP^{+}}}$$

The values of *P*_*NPQ*_ are assumed to range from 0 (inactive) to 1 (fully active). *P*_*NPQ*_ is modeled with a saturating curve whose formation depends on *CEF*. In fact, as previously described, the activation of the proteins related to NPQ is triggered by a strong change in luminal pH, which occurs when a decoupling of the light reactions with the dark reactions generates an excess in energy that is exploited by the cyclic electron transport (*CEF*) to produce a decrease in the luminal pH as well as a production of ATP.

Finally, the energy flowing from PSII to PSI (*ETR*) is used to reduce NADP^+^ to NADPH whose dynamics are described as follows:


(4) $$\frac{dNADPH}{dt}=\overset{ETR}{\overbrace{v_{ETR}\cdot E_{PSII}\cdot NADP^{+}}}\cdot \eta_{NADP^{+}}-\overset{A}{\overbrace{v_{C}\cdot R\cdot NADPH}}\cdot \eta_{NADPH}$$

(5) $$\frac{dNADP^{+}}{dt}=-\frac{dNADPH}{dt}$$

The two η parameters represent the efficiencies of the linear electron transport in producing *NADPH* (η_*NADP*^+^_) and consuming *NADP*^+^ (η_*NADPH*_). In particular, the parameter η_*NADPH*_ regulates the velocity of the Calvin Cycle and, therefore, can be related to the RuBP regeneration. Equation 6 describes the dynamics of *NADP*^+^ just as opposed to the one of *NADPH*. Carbon assimilation (*A*) is, therefore, regulated by the rate of carboxylation (*v*_*C*_) mediated by Rubisco (*R*). The dynamics of Rubisco activation is described by the following equation:


(6) $$\frac{dR}{dt}=\overset{Rubiscoactivation}{\overbrace{v_{R}\cdot \left(1-R\right)}}\cdot \text{min}\left(d,\Delta pH\right)$$

with


$$△pH=\frac{NADPH}{NADP^{+}}$$

*R* is modeled with a saturating curve whose formation depends on the change of transmembrane ΔpH, which can be accounted as the ratio between NADPH and NADP^+^ (derived by Morales et al., 2018). The parameter *d* represents the maximum value of △pH allowing for a smoother curve in the first phases of the activation of the Rubisco.

The description of the six state variables and the parameters with the relative units are found in Table 1. The model allows the characterization of three quantities measured in gas exchange and fluorescence analysis: *ETR*, *A*, and *NPQ*. These three quantities are fluxes (μmol m^–2^ s^–1^) and can be derived from the described equations: *ETR = v*_*ETR*_⋅*E*_*PSII*_⋅*NADP*^+^ from Equation 1, *NPQ = v*_*NPQ*_⋅*Q* from Equation 2, and *A = v*_*C*_⋅*R*⋅*NADPH* from Equation 4.

### Sensitivity Analysis

A local sensitivity analysis (Norton, 2015) was performed to analyze the model behavior under parameter perturbation. The normalized sensitivity index is calculated by changing each parameter of ± 5% while keeping all the other constant. The equation for the sensitivity index is the following:


(7) $$SSE_{i,△}=\sqrt{\frac{1}{3\cdot n}\sum_{j=1}^{3}\sum_{i=1}^{n}{\left(\frac{X^{j}\left(p_{1},p_{2},\ldots ,p_{i}+△,\ldots ,p_{k}\right)-X^{j}\left(p\right)}{\max\left(X^{j}\left(p\right)\right)-\text{min}\left(X^{j}\left(p\right)\right)}\right)}^{2}}$$

where *SSE*_*i*,△_ is the standardized elementary effect of the parameter *p_i_* with Δ (± 5%) perturbation on model outputs and *k* is the number of parameters (equal to 13); *X^j^*(**p**) are the simulated values of the *j*-th quantity considered (i.e., *NPQ*, *ETR*, and *A*) without any parameter perturbation (as given in Table 1); and *n*is the number of samples per observed quantity (equal for three quantities considered).

## Results

### Experimental Data

**FIGURE 2:**
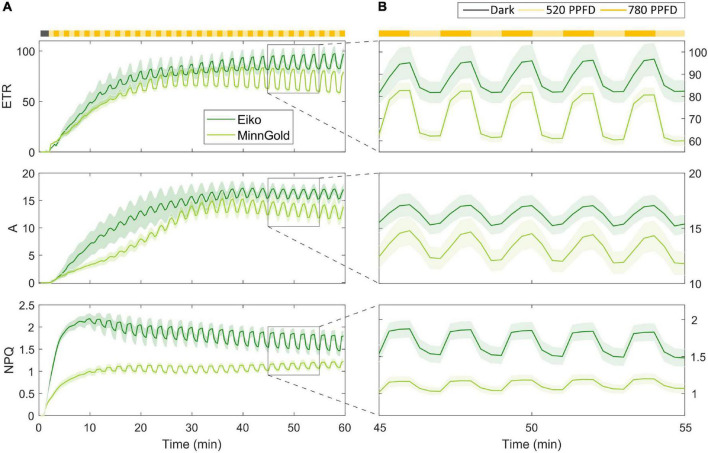
Fluorescence data coupled with gas exchange data in the fluctuating light regime for the two varieties Eiko (indicated in dark green) and MinnGold (indicated in light green). The light intensity was fluctuating every min from 780 (indicated in dark yellow) to 520 μmol m^– 2^ s^– 1^ (indicated in light yellow). **(A)** Data taken from all the experimental periods (60 min) **(B).** Focus on the fluctuations from 45 to 55 min.

The model has been tested on fluorescence data coupled with gas exchange data in the fluctuating light regime for the two varieties, namely, Eiko and MinnGold. As described in the “Materials and Methods” section, the leaf was kept in the dark and then illuminated with fluctuating light at 520 and 780 μmol m^–2^ s^–1^ for 60 min. In particular, the changes in electron transport (*ETR*), carbon assimilation (*A*), and *NPQ* were recorded (Figure 2).

After illumination, *ETR* and *A* show an initial slow photosynthetic induction (slower for MinnGold) mainly caused by the activation of the enzyme Rubisco (Soleh et al., 2016; Taylor and Long, 2017) in which the fluctuations in light are not causing, initially, corresponding fluctuations in these quantities. When Rubisco is fully activated, a steady-state is reached, and the fluctuations become more evident and constant throughout the experimental period (the last 30 min). Regarding *NPQ*, a faster rise of this quantity is evident with an increase in the amplitude of fluctuations in time connected to the increased level of NPQ-related proteins. Figure 2B focuses on a smaller experimental period when a steady-state is already being reached (from 45 to 55 min). MinnGold results are more responsive to fluctuations of light, in the sense that the changing in light intensity is causing higher amplitudes of oscillations in *ETR* and, to a smaller extent, to *A*. In *NPQ*, it can be observed the opposite behavior, with fluctuations of light causing smaller amplitudes of oscillations.

### Model Fitting

**FIGURE 3:**
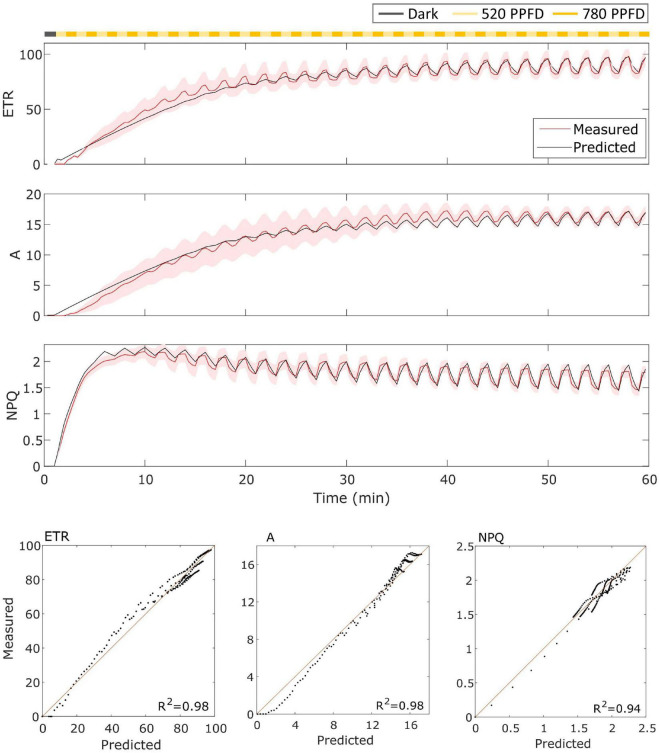
**(Top)**. Eiko data (indicated in red) compared to model results (indicated in black) in the fluctuating light regime. The data are shown as means of three replicates (indicated as a red continuous line) and their standard error (indicated as the red shaded area around the mean value). **(Bottom)**. Parity plots for ETR, *A*, and NPQ with related *R*^2^.

For the wild type Eiko (Figure 3), the model accurately represented the measured dynamics with an *R*^2^ of 0.98 for *ETR* and *A* of 0.94 for *NPQ*. In this case, the model captured both the slow induction dynamics and the fast fluctuating dynamics.

**FIGURE 4:**
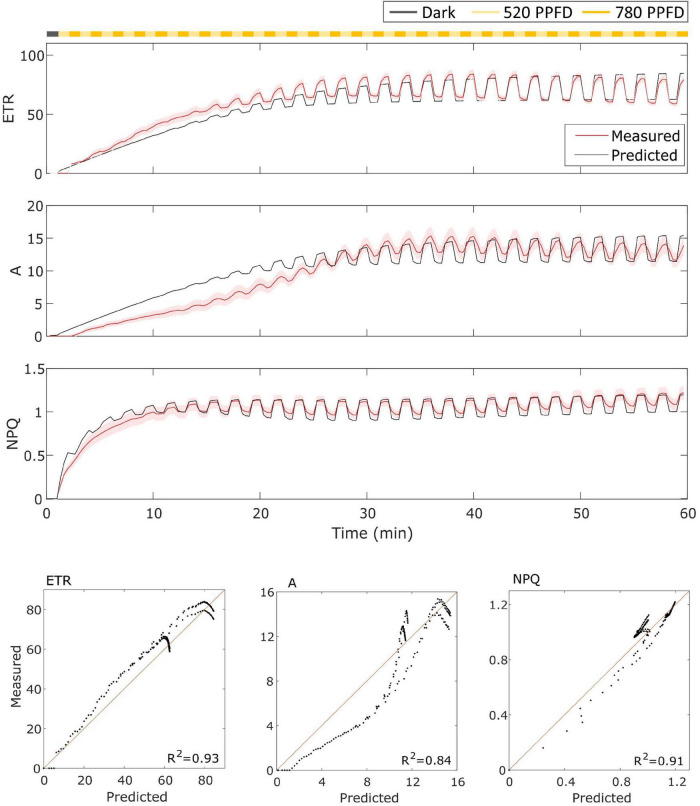
**(Top).** MinnGold data (indicated in red) compared with model results (indicated in black) in the fluctuating light regime. The data are shown as means of three replicates (indicated as a red continuous line) and their standard error (indicated as the red shaded area around the mean value). **(Bottom).** Parity plots for ETR, *A*, and NPQ with related *R*^2^.

In the case of MinnGold (Figure 4), the model performed well for both *ETR* and *NPQ* (*R*^2^ = 0.93 and 0.91, respectively), whereas it did not capture the slow induction found in *A*, still having a good *R*^2^ = 0.84.

### Validation

**FIGURE 5:**
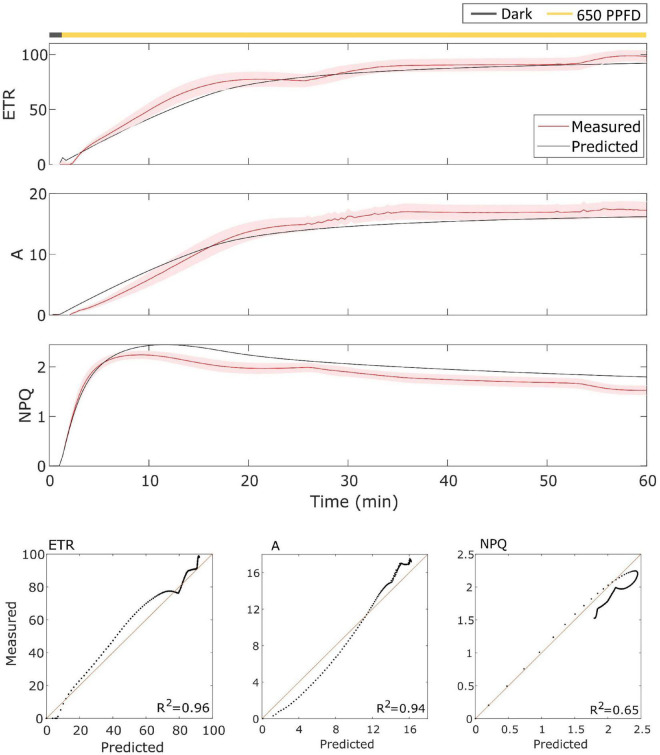
**(Top).** Eiko data (indicated in red) compared with model results (indicated in black) in constant light. The data are shown as means of three replicates (indicated as a red continuous line) and their standard error (indicated as the red shaded area around the mean value). **(Bottom).** Parity plots for ETR, *A*, and NPQ with related *R*^2^.

**FIGURE 6:**
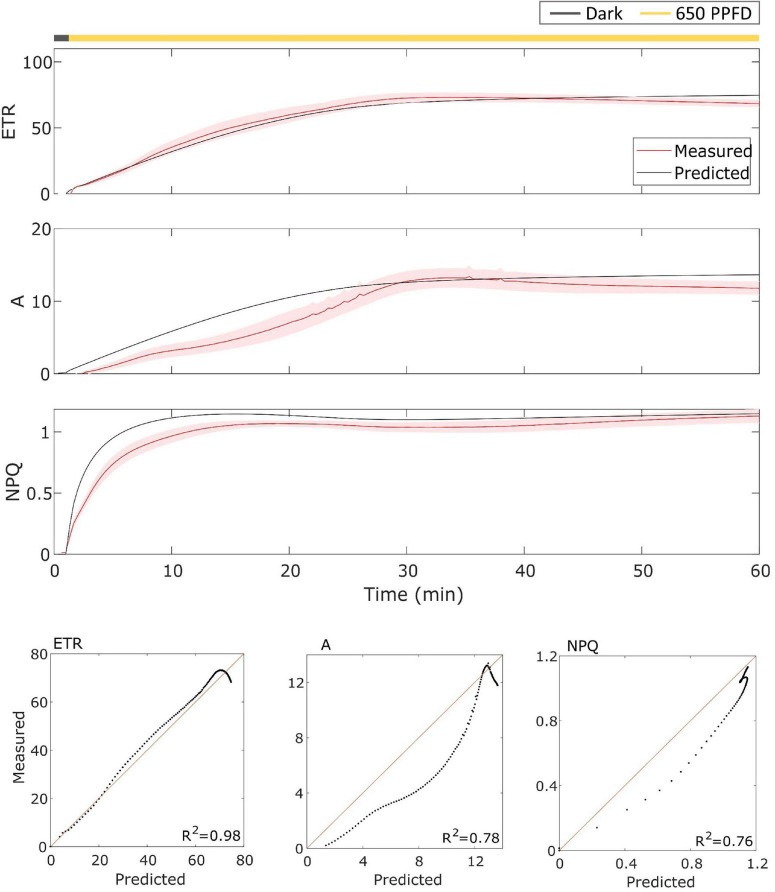
**(Top).** MinnGold data (indicated in red) compared with model results (indicated in black) in constant light. The data are shown as means of three replicates (indicated as a red continuous line) and their standard error (indicated as the red shaded area around the mean value). **(Bottom).** Parity plots for ETR, *A*, and NPQ with related *R*^2^.

The model has been then validated on gas exchange data in the constant light regime. To validate it, the model has been tested over the data using the parameters found for the fluctuating light protocol in Eiko (Figure 5) and MinnGold (Figure 6 and Table 1). The model performed well also in these conditions, in particular for *ETR* (*R*^2^ = 0.96 and 0.98 for Eiko and MinnGold, respectively) and *A* (*R*^2^ = 0.94 and 0.78), with a slight underperformance for *NPQ* (*R*^2^ = 0.65 and 0.76).

### Sensitivity

**FIGURE 7:**
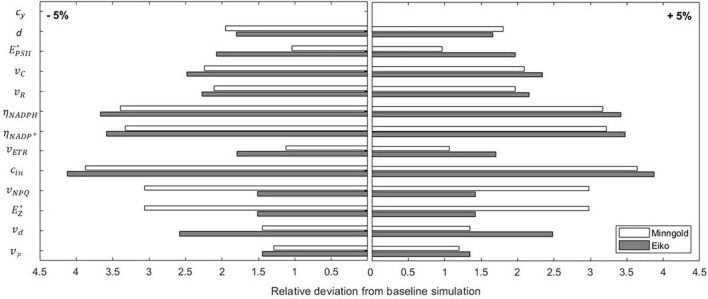
Sensibility analysis of the model parameters for both MinnGold and Eiko. The parameters have been perturbed of ± 5% around the value in Table 1, and the relative deviation from baseline simulation of the model output was calculated.

The local sensitivity analysis (Equation 8) allowed identifying the parameters whose change mainly affected the three quantities considered (i.e., *A*, *ETR*, and *NPQ*). By changing the parameters by 5%, the outcome of the model never deviates more than 4% from the baseline simulation (Figure 7). This means that the model is robust and not much dependent on the changes in the parameters; furthermore, no matter if the percentage change is positive or negative, the outcome is the same.

The sensitivity also showed differences in MinnGold and Eiko. In both cases, the parameter more sensible to changes is *c*_*in*_, the parameter identifying the energy input in PSII and, therefore (with α, the absorbance coefficient), the energy entering the photosystem. Differences among MinnGold and Eiko can be found for $E_{PSII}^{*}$, the carrying capacity of the PSII. A small difference for the two species can also be found for the parameters *v*_*NPQ*_, $E_{Z}^{*}$, and *v_d_*.

### Theoretical Evaluation of the Model

**FIGURE 8:**
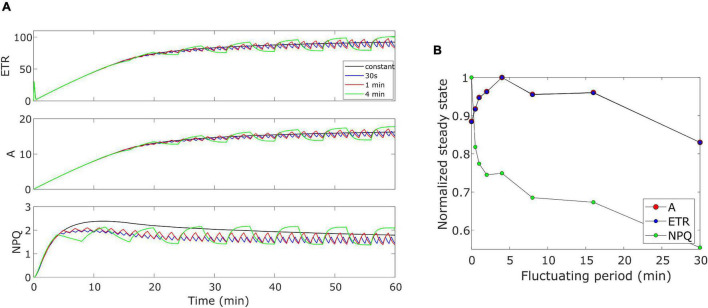
**(A)** Varying fluctuating period of light in Eiko. Light is fluctuating every 30 s, 1 min (as in the experiment), and 4 min. **(B)** Effect of varying fluctuating light on the steady-state variable (cumulative value after 40 min). 0 fluctuating period means constant light.

The model was further validated by performing some theoretical simulations by considering Eiko parameters (Table 1). We evaluated how the three quantities (i.e., *ETR*, *A*, and *NPQ*) would behave when changing the period of the fluctuating light. Figure 8A shows an example of the effect of three different fluctuating periods (i.e., 30 s, 1 min, and 4 min fluctuating period with duty cycle equal to 0.5) when compared with the constant light regime. When calculating the cumulative values at steady-state (after 40 min), *A* and *ETR* resulted higher than those for constant light (fluctuating period equal to zero) when light fluctuates with a period higher than 30 s and lower than 20 min (Figure 8B).

Nevertheless, we have the opposite behavior for *NPQ*. We also calculated modeled cumulative steady-state values with MinnGold parameters (Supplementary Figure 3). Steady-state values of *ETR* and *A* decreased as the fluctuation period increased, except for short fluctuating periods in which they increase (of the same order as Eiko, Figure 8B). Nevertheless, in this case, we found fluctuations causing a much smaller change in *NPQ* steady state.

We finally performed simulations with higher fluctuations intensity, with the same fluctuating period (1-min period) (Supplementary Figure 4). In this case, the constant regime results always higher than the fluctuating regime; therefore, the higher the fluctuation amplitude, the lower would be the steady-state value.

## Discussion

### Model Assumptions

Here we presented a leaf level System Dynamics photosynthesis model based and validated on an experiment performed on two soybean varieties, namely, the wild type Eiko and the chlorophyll-deficient mutant MinnGold, grown in constant and fluctuating light conditions.

The model was developed to reflect the assumptions of the experimental conditions. Leaves were exposed to optimal CO_2_ conditions and average light intensity; therefore, we assumed no limitation due to stomatal conductance. Two main conditions are investigated: (1) the photosynthetic induction during the dark-light transition and (2) the fluctuations of light maintaining the system in a continuous non-steady-state condition. One of the main results of this work is found in the modeling of the cyclic electron transport, which is thought to be fundamental in the triggering of *NPQ* when *ETR* is still limited by the downstream reactions of the Calvin Cycle (Cornic and Baker, 2012; Yamori and Shikanai, 2016). In fact, the dissipation of energy through NPQ is possible when zeaxanthin forms a quenching complex with PSII and PsbS proteins that have been protonated. These two processes are both triggered by a change in ΔpH which, when *ETR* is limiting, is caused by the cyclic electron transport. The fact that *NPQ* activation is possible also when *ETR* is not fully active is evident from the data, both in the long-term and in the short-term. Figure 2A in fact shows that *NPQ* reaches a steady-state much faster than *ETR* and *A* during the dark-light transition. This is also evident in the short term: in fact, during the fluctuations of light (Figure 2B) at steady-state, *NPQ* is still found to be faster than the other quantities in reaching the steady-state associated with the specific light intensity.

We only modeled the qE component of NPQ due to the time scale of the measurements, though also qT and qI (to a lesser extent, due both to the time scale and light intensity) can act, as modeled by Ebenhöh et al. (2014). Furthermore, the generation of the energy-dependent NPQ is modeled as a single process. Though it is known that at least two different quenching sites contribute to NPQ (Nilkens et al., 2010): the PsbS-dependent located in detached antenna complexes (LHCII) and the zeaxanthin-dependent (qZ) located in smaller antenna proteins that remain attached to the reaction center (Holzwarth and Jahns, 2014). Both processes are pH-dependent (Matuszyńska et al., 2016) and operate independently from each other. In our case, for the sake of simplicity, we have combined them in the variable named *P*_*NPQ*_ (Equation 3).

Finally, the local sensitivity analysis has demonstrated that the model is robust to changes in parameter values since a small change in the input parameters does not produce unexpected and unrealistic changes in the model outcome (Figure 7; Saltelli et al., 2004). Furthermore, it enables to identify the parameters which mostly influence the model outcome (in this case, *c*_*in*_ and $E_{PSII}^{*}$) and that are species-specific.

### Model Performance

The model was able to simulate the experimental data reasonably well both in constant and fluctuating light conditions and in both soybean varieties with *R*^2^ ranging from 0.65 to 0.98 (Figures 3–6). Nevertheless, two main observations were not well-fitted by the model, in addition to some finer details.

First, in MinnGold, it is found a decoupling of *A* and *ETR* in the velocity of induction in both fluctuating (Figure 4) and constant (Figure 6) light conditions. At a steady-state, the two processes are known to be coupled, since the electron chain starts when electrons are reducing NADP^+^, which are in turn mainly produced by the Calvin Cycle. In our model, in fact, higher ETR generates more NADPH (Equation 4), which in turn activates carbon assimilation (A). This framework, though, does not take into account photosynthetic control mechanisms *via* Cyt b6f activity (Johnson and Berry, 2021) as well as various photoprotective mechanisms to prevent photoinhibition (Yamori, 2016). Furthermore, it is known that electrons can also be transferred to other enzymes involved in the regulation of carbon metabolism as well as in nitrogen and sulfur metabolism (Cornic and Baker, 2012). Nevertheless, we think that this decoupling is due to the fundamental assumptions for calculating *ETR* based on fluorescence. In fact, it is assumed that the fraction of absorbed light that is directed to PSII and the leaf absorbance are constant in the *ETR* (Baker, 2008), whereas this might not be generally true during the induction dynamics and for Chl-deficient varieties. Since this would need a further discussion and a focus on the nature of this result, we did not aim to capture this dynamic.

The second observation differing from the model is found in *NPQ* steady-state when calculated in constant light. The model in fact overestimated the steady-state values in both Eiko and MinnGold (Figures 5, 6). This might be an adaptation strategy. Since the constant regime is less stressful for the plants, it might be that less energy needs to be dissipated as heat. Since the model was calibrated to the fluctuating light data, a stressful condition, it might be that the parameters regulating *NPQ* are set higher than necessary for the constant regime. To capture this difference, it would be probably necessary to introduce a framework regarding the adaptation of the plants based on their growing conditions.

Then, concerning only the fluctuating light part, the model simulated signal changes slower than those measured for Eiko (Figure 3) and faster for MinnGold (Figure 4) for all the three considered quantities. Regarding NPQ, the observed deviations can be attributed to the simplified modeled mechanisms of generation of NPQ, which in turn regulates ETR. Indeed, two different processes have been identified to contribute to NPQ induction and relaxation dynamics: a fast one that requires the generation of a proton gradient sensed by PsbS and a slow one that is activated by low luminal pH that triggers de-epoxidation of violaxanthin to zeaxanthin (Matuszyńska et al., 2016). These two different quenching sites (PsbS-dependent, Q1; and zeaxanthin-dependent, Q2) determine four different NPQ states of PSII, which can be distinguished upon illumination of dark-adapted leaves (the so-called 4-state 2-site model of NPQ; Holzwarth and Jahns, 2014). These four states are characterized by the different velocities of activation/deactivation of the two quenching sites. In these experiments, we expected the following: dark-adapted leaves are in the state I in which Q1 and Q2 sites are inactive; as soon as the light is turned on, the Q1 site is activated by the ΔpH (state II); after 10–20 min upon illumination, Q2 is activated by the low pH (state III); once established, Q2 remains active for a longer time; therefore, high-frequency transitions from high to low light would relax only Q1. In fact, relaxation dynamics of Q1 occur in the order of 1–2 min, that is, the same as the frequency range used in the fluctuations of light in the experiment.

The oversimplified model combines the two kinetics occurring during photosynthetic induction and light fluctuations, causing the observed deviations between experimental data and model simulations (Figures 3–6). As it has been modelled, NPQ responses during the first induction phase and at steady state with light fluctuations are always slower in Eiko (Figure 3) and faster in MinnGold (Figure 4) compared to experimental data. Therefore, it would be necessary to explicitly include a second variable to represent both PsbS- and zeaxanthin-related processes in further developments of the model in order to describe all the different NPQ states and to capture more finely these described dynamics. Finally, the theoretical analysis of the model allowed making some relevant conclusions. When calculating the cumulative values at steady-state using Eiko’s parameters in respect to different fluctuating light periods (Figure 8B), we found *A* and *ETR* steady-state values increased by reaching a maximum at 5 min fluctuating period and then to decrease for fluctuating periods longer than 20 min. Therefore, it seems that a certain range of fluctuations of light is favorable for the cumulative steady-state carbon assimilation, coherent with the observations of Graham et al. (2017). This behavior is confirmed with MinnGold parameters (Supplementary Figure 3), but in this case, we found much smaller changes in *NPQ* steady-state, meaning probably that *NPQ* relaxation dynamics in MinnGold are faster than those in Eiko, this being opposed to what proposed by Sakowska et al. (2018). More in general, the understanding of the *NPQ* influence in regulating dynamic photosynthesis is still controversial. Two recent articles have in fact found an opposite trend in biomass accumulation when accelerating *NPQ* relaxation time (Kromdijk et al., 2016; Garcia-Molina and Leister, 2020).

By being a theoretical mathematical model, when referring to the values of the parameters, it is relevant to look at the relative differences among the varieties, whereas the absolute values might not be always coherent with the biology. This is though due to the calibration procedure in finding local minima; therefore, other combinations of parameters are possible. Nevertheless, when looking at Table 1, almost all parameters are found to be comparable among the two varieties, confirming the robustness of the model. Only three parameters, namely, $E_{PSII}^{*}$, *v*_*ETR*_, and *v_d_*, differ. $E_{PSII}^{*}$ identifies how much energy can be held by the PSII and it represents the number of chlorophyll molecules in the chloroplast, which is known to be different for Eiko and MinnGold (Slattery et al., 2017; Sakowska et al., 2018). This parameter value, therefore, is reasonably much higher in Eiko than in MinnGold. Nevertheless, *v*_*ETR*_ and *v_d_* are the velocities of activation of *ETR* and *NPQ*, respectively, and are higher in MinnGold. This can be explained by the fact that even if MinnGold has a much lower number of chlorophyll molecules, this number is sufficient to have a responsive *ETR* and *NPQ*, which can sustain comparable carbon assimilation. In particular, both the model and the experimental data show MinnGold to be even more responsive to fluctuations of light; in fact, the fluctuating light causes higher oscillations in *ETR* and *A* (Figure 2).

### Comparison With Other Models

The model presented focuses on the limitations imposed by light reactions, due to the nature of the experiment conducted; therefore, the downstream regulation is much simplified. The model, therefore, is neither as comprehensive as preceding models (Morales et al., 2018; Bellasio, 2019) nor as detailed as other molecular models (Zhu et al., 2013; Nedbal and Lazàr, 2021), but, even with its limitations, it demonstrated that a macro representation of the processes is still able to capture well the dynamics found in photosynthesis and helps in unraveling gas exchange and fluorescence data. Furthermore, the model focuses on low-intensity fluctuations of light in order to avoid photoinhibition and to stress photosynthesis due to fluctuations of light only. Nevertheless, the photoinhibitory component of NPQ (qI) or ROS production could be added in the further development of this model to deal with higher light intensities.

The main drawbacks of the model are due to the combination of the two NPQ kinetics (PsbS- and zeaxanthin-dependent), causing the presence of deviations between experimental data and model outcomes during the induction phase and the fast fluctuations of light at steady-state. The representation of both kinetics by two separate variables would allow a more appropriate description of the two existing dynamics operating at different time scales, with expected higher accordance of the model simulations with data.

Nevertheless, since the limited number of equations and related parameters, this model could become one of the building blocks of a photosynthesis model at higher scales, both leaf and canopy. Since there are already other system dynamics models, following the same procedure, focused on the dark reactions (Kirschbaum et al., 1997) and on stomatal conductance (Vialet-Chabrand et al., 2016), it would be interesting to combine the presented model with these existing ones to simulate the most dynamic environmental conditions and allowing for an upscaling. In fact, even if relevant canopy level photosynthesis models exist (van der Tol et al., 2009; Song and Zhu, 2012), none, to our knowledge, aims to capture the responses of photosynthesis to dynamic environmental conditions, since it would be too complicated with the available tools. In fact, by focusing on non-steady-state conditions, we think that it is necessary to provide a more simplified representation of the system: we aim not to explain the measurements but to create a tool able to reproduce the observed dynamics, and due to its simple formulation, to apply the model to bigger scales.

## Conclusion

In this study, we presented and validated a new system dynamic model based on the light reactions of photosynthesis. Since plants are normally dealing with dynamic environmental conditions, it should be considered to introduce into models such processes in photosynthesis that are usually discarded in steady-state models, such as the cyclic electron transport (that we represented in this model), and many other processes, such as the water-water cycle, the malate shuttle, and the other components of NPQ (Yamori, 2016), which become limiting when conditions are unsteady.

To further address finer details of the observed dynamics, it would be necessary to introduce further experimental analysis to unravel the dynamics of LHCII, the PsbS protein, and thylakoid lipids (Nilkens et al., 2010; Jahns and Holzwarth, 2012; Ruban and Wilson, 2021) and to distinguish between various components of excess energy dissipation (Holzwarth and Jahns, 2014; Chukhutsina et al., 2019). In the case of MinnGold, a quantitative characterization of photosynthetic pigment analyses is necessary since only qualitative data are now available. These data would allow further refinement of the model. Furthermore, a more detailed description of the different NPQ dynamics will allow a better performance of the model.

Finally, even if the proposed model is at the leaf level, due to its simplicity, it could easily be one of the building blocks of a more comprehensive photosynthetic model at a plant or even canopy scale. Upscaling both the models and the experiments is fundamental since translating these short-term leaf scale results into the field is not straightforward (Kaiser et al., 2018; Matsubara, 2018). In particular, in this case, we found fluctuations of light to not interfere in the performance of MinnGold in such a short-term analysis even if it is hypothesized that they might have an effect in the long-term. Therefore, canopy level data and models become fundamental in unraveling dynamic photosynthetic processes.

## Data Availability Statement

The raw data supporting the conclusions of this article will be made available by the authors, without undue reservation.

## Author Contributions

NS and FC wrote the manuscript. NS and GA performed the experiments. NS, FG, FC, and SM designed the model. NS, FC, and FG wrote the equations. NS implemented the model. SM and AP supervised the work. All authors agreed on the final version of the manuscript.

## Conflict of Interest

The authors declare that the research was conducted in the absence of any commercial or financial relationships that could be construed as a potential conflict of interest.

## Publisher’s Note

All claims expressed in this article are solely those of the authors and do not necessarily represent those of their affiliated organizations, or those of the publisher, the editors and the reviewers. Any product that may be evaluated in this article, or claim that may be made by its manufacturer, is not guaranteed or endorsed by the publisher.
