# Supplementary material for: A System Dynamics Approach to Model Photosynthesis at Leaf Level Under Fluctuating Light
Source: Front Plant Sci. 2022 Jan 28;12:787877. doi: 10.3389/fpls.2021.787877 (PMC8833254; doi:10.3389/fpls.2021.787877)
Supplement: Supplementary file 1 [file Data_Sheet_1.docx]

Supplementary Material

## Supplementary Figures

##
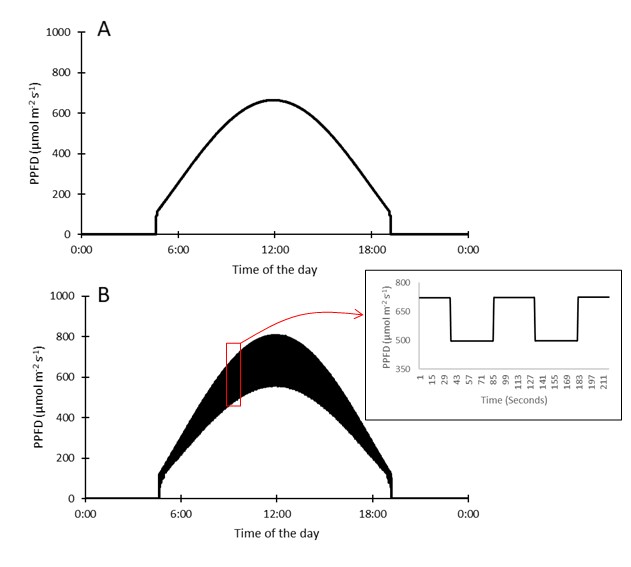


**Supplementary Figure 1.** Growth conditions in non-fluctuating (A) and fluctuating (B) light. The insert in B shows the variations of light intensity within 3 minutes.

**
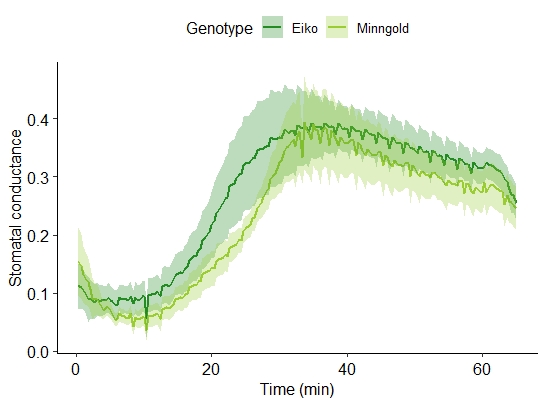
**

**Supplementary Figure 2.** Changes in stomatal conductance during photosynthetic induction (fluctuating protocol) in MinnGold and Eiko.


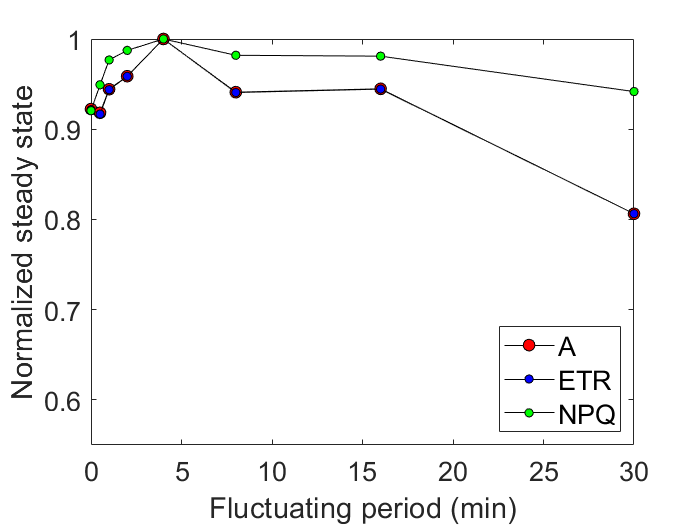


**Supplementary Figure 3.** Effect of varying fluctuating light period on steady state in MinnGold. The figure shows the cumulative value after 40 minutes.


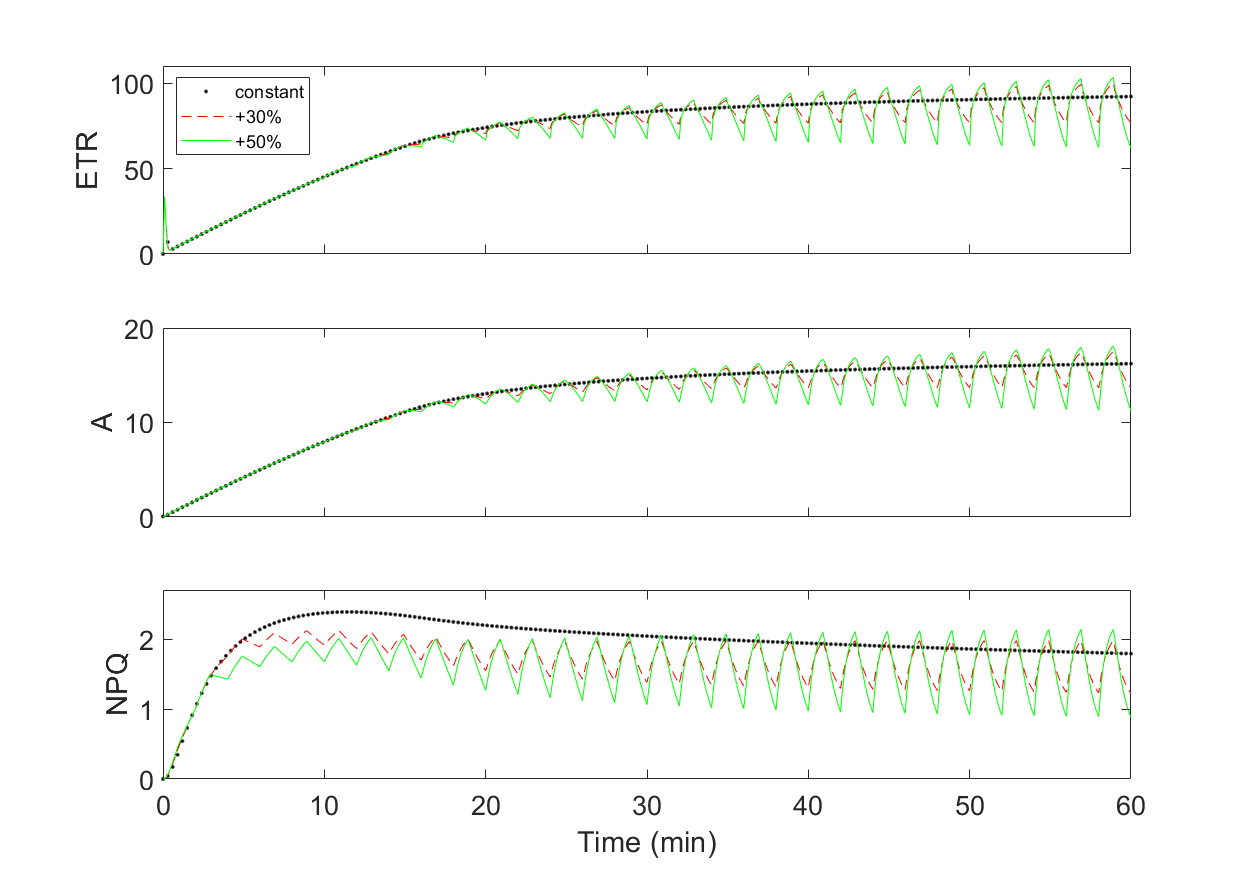


**Supplementary Figure 4.** Varying fluctuating intensity of light in Eiko. Light was either kept constant at 650 PPFD or fluctuating every minute at two different intensities: 650 $\pm$ 30% and 650 $\pm$ 50%
